# Celiac Disease, Beyond the Bowel: A Review of Its Neurological Manifestations

**DOI:** 10.7759/cureus.20112

**Published:** 2021-12-02

**Authors:** Saawan C Patel, Devarashetty Shreya, Diana I Zamora, Gautami S Patel, Idan Grossmann, Kevin Rodriguez, Mridul Soni, Pranay K Joshi, Ibrahim Sange

**Affiliations:** 1 Internal Medicine, Pramukhswami Medical College, Karamsad, IND; 2 Internal Medicine, Gandhi Medical College, Secunderabad, Hyderabad, IND; 3 General Medicine, Universidad de Ciencias Médicas Andrés Vesalio Guzman, San José, CRI; 4 Research, Medical University of Silesia in Katowice Faculty of Medical Sciences, Katowice, POL; 5 Research, Universidad Americana (UAM) Facultad de Medicina, Managua, NIC; 6 Research, Shri Lal Bahadur Shastri Government Medical College, Mandi, IND; 7 Department of Medicine, Byramjee Jeejabhoy Medical College, Ahmedabad, IND; 8 Research, Karamshi Jethabhai Somaiya Medical College, Mumbai, IND

**Keywords:** gluten intolerance, gluten-related neuropathology, extra-intestinal manifestations of celiac disease, gluten neuropathy, gluten encephalopathy, gluten ataxia, neurological features of celiac disease

## Abstract

Celiac disease (CD) is a multi-systemic autoimmune condition that causes a hyperinflammatory response when gluten is ingested. There has been a shift in the clinical presentation of CD from a mere malabsorption disorder to an autoimmune condition that affects multiple organ systems, which could increase the rate of hospitalizations and a decreased quality of life. This article has compiled various studies that have explored the neurological manifestations of celiac disease, their epidemiology, possible pathogenic mechanisms, diagnosis, and treatment. The most common neurological conditions include gluten ataxia (GA), gluten neuropathy, gluten encephalopathy, and epilepsy which usually present as sporadic diseases which are difficult to diagnose in the absence of gastrointestinal (GI) symptoms. The treatment for most of these conditions is a gluten-free diet (GFD) regardless of GI involvement.

## Introduction and background

**Figure 1:**
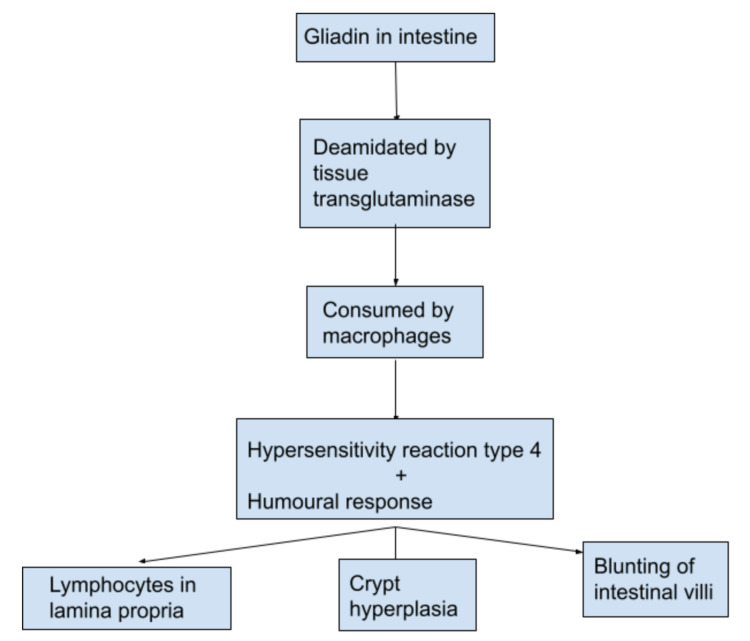
Pathogenesis of celiac disease

Celiac disease (CD), previously known as celiac sprue, is an autoimmune condition in which genetically predisposed individuals develop an immunologic reaction to ingested gluten, a protein found in barley, wheat, and rye, destroying the intestinal villi [1]. It is a condition that is quite commonly underdiagnosed due to its variable clinical presentations, wide age group, and unclear pathogenesis. About 1% of the world's population is affected by CD, most commonly in New Zealand, Argentina, Hungary, Sweden, Finland, India, and Egypt [2,3]. CD exhibits a higher incidence in the paediatric age group and has a slight proclivity towards females compared to males [4]. As per the latest medical literature, it has been well established that CD is more frequent in individuals who already have a diagnosed first-degree relative with a higher prevalence if the family has two or more siblings affected [3,4]. The presence of the human leukocyte antigen (HLA)-DQ2 and HLA-DQ8 allele has been well documented in 90% of the patients diagnosed with CD [5]. Gliadin, a protein found in gluten, is the major pathogenic component in CD [6]. It is deamidated by tissue transglutaminase (tTG), making it available for consumption by antigen-presenting cells (APCs). This, in turn, leads to T-cell mediated hypersensitivity reaction (type 4) and a humoural response resulting in histologic changes in the small intestine, such as lymphocytes in the lamina propria, crypt hyperplasia, and blunting of the intestinal villi (Figure 1) [2].

As a result, flatulence, bloating, chronic diarrhea, alternating bowel habits, weight loss, and steatorrhea are considered symptoms of classical CD according to Oslo's definitions [7]. However, the term 'classical' is misleading, as 66% of the patients with symptomatic CD express the non-classical phenotype [7]. Due to a widespread multisystem involvement, the CD is accompanied by a vast spectrum of extraintestinal involvement leading to haematological, dermatological, musculoskeletal, and neurological manifestations [8]. With a growing proportion of patients with the non-classical phenotype, the clinical picture of CD has evolved through the years. The work-up usually begins with the detection of antibodies in the serum, such as IgA anti-tTG antibody IgA because of its excellent sensitivity (93%) and specificity (95%) [9]. A biopsy may be performed in clinical cases with high suspicion if the serologies are negative [9]. A gluten-free diet remains the mainstay of treatment; however, there are ongoing clinical trials for glutenases, steroids, and immunosuppressants in the form of non-dietary therapy [9,10]. It is challenging to screen and diagnose due to a complex presentation coupled with an unpredictable age of onset. In the United States, it is estimated that 90% of patients with CD are undetected, and the cases that are diagnosed are due to at-risk group screening rather than clinical case discovery [11,12]. While most of the patients suffering from CD have symptoms of malabsorption, there is a wide variety of extraintestinal manifestations that exhibit complex overlapping symptomatology that makes the diagnosis difficult and challenging [8]. This review article aims to: (i) underline the pathogenic mechanism of the involvement of CD and its neurological manifestations; (ii) establish a clinical relationship between CD and its neurological manifestations; (iii) explore the existing screening and upcoming management guidelines of CD.

## Review

The most common neurological manifestations of CD include gluten ataxia (GA), gluten neuropathy, and epilepsy [13]. Each of them will be discussed in the subsequent text.

Gluten ataxia

It is defined as an autoimmune condition in which gluten consumption damages the cerebellum causing difficulties in gait control, muscle coordination, and reduced fine control of voluntary movements [13]. It is present in about 6% of the patients with CD and in 40% of the patients with CD who have neurological symptoms [14]. GA is the most common cause of sporadic ataxia (25%) [15]. A study conducted by Hadjivassiliou et al. in 2016 followed 1500 patients with ataxia over 20 years with subsequent clinical assessment every six months and found that 1200 of the 1500 patients had sporadic ataxia. Out of 1200, 300 patients were diagnosed with gluten ataxia (Table 1) [15].

**Table 1:** Summary of included studies revealing the pathogenesis, clinical features, and treatment of gluten ataxia GA, gluten ataxia; CD, celiac disease; MRS, magnetic resonance spectroscopy; anti-TG6, anti-tissue transglutaminase; GFD, gluten-free Diet; NAA/Cr, N-acetyl aspartate/creatine

References	Design	Population	Method	Findings	Conclusion
Hadjivassiliou et al. [15]	-	Sheffield Ataxia Centre, United Kingdom	1500 patients with cerebellar ataxia	The patients were assessed for 20 years on a 6-monthly basis, and 25% of the patients with sporadic ataxia had GA.	GA should be suspected in a patient with cerebellar ataxia in the absence of family history.
Briani et al. [20]	-	Department of Neuroscience at University of Podova, Italy	71 biopsy confirmed patients with CD	The patients were tested for neurological symptoms and anti-neural antibodies. 16 patients had neurological symptoms, but 30 patients exhibited antibody reactions to neural antigens.	Neurological symptoms and anti-neural antibodies are unrelated.
Hadjivassiliou et al. [21]	Prospective cohort	Sheffield, United Kingdom	100 newly diagnosed patients with CD by duodenal biopsy and gastroscopy	Patients with neurological symptoms underwent MRS and serum anti-TG6 test. 73% of the patients with an abnormal MRS of the cerebellum, ataxia, or both had anti-TG6 antibodies.	The presence of anti-TG antibodies may be used for screening and diagnosis of GA.
Hadjivassiliou et al. [22]	Prospective, single-center, observational case series	Sheffield, United Kingdom	117 patients with GA	To assess the effect of GFD on MRS of the cerebellum by calculating NAA/Cr area ratio. The NAA/Cr area ratio increased in 98% of the patients on a GFD.	GFD can be used effectively to treat GA even in the absence of enteropathy.

With the average age of onset being 48 years, patients usually present with pure cerebellar ataxia with lower limb involvement in 90% of the patients and upper limb involvement in 75% of the patients. There is gaze-evoked nystagmus in addition to other ocular signs of cerebellar insult in 84% of the patients [16]. Evidence also suggests that patients need not necessarily have GI symptoms as a prerequisite to displaying gluten ataxia as only 40% of patients with GA have evidence of enteropathy [17]. A study conducted in 1998 by Hadjivassiliou et al., included 28 patients with gluten ataxia, out of which 16 had no GI symptoms, and only 11 demonstrated features of CD on distal duodenal biopsy [18].

With regards to the pathogenesis, patchy loss of Purkinje cells has been observed on post-mortem examination of patients who had GA [19]. There was astrocytic gliosis, diffuse infiltration, and perivascular cuffing, predominantly by T-lymphocytes of the cerebellar white matter [19]. The presence of cerebellar damage has been established, but the mechanism seems to be unclear as evidence suggests no correlation between an autoimmune response and neurological dysfunction (Table 1) [20]. According to a study conducted in 2008 by Briani et al., a group of 71 biopsy-confirmed CD patients was screened for serum antibodies to neural antigens and neurological deficit. It was found that only 16 patients had neurological symptoms, but 30 patients exhibited antibody reactions to neural antigens thereby, indicating that anti-neural reactivity and neurologic impairment do not appear to be linked because anti-neuronal antibodies were present in the absence of neurological symptoms [20]. There is, however, recent evidence to suggest that the presence of IgA anti-transglutaminase 6 (TG6) antibodies has a significant link to brain atrophy in patients with CD. A prospective cohort study conducted in 2019 by Hadjivassiliou et al. followed 100 newly diagnosed CD patients by duodenal biopsy and gastroscopy, who underwent magnetic resonance spectroscopy (MRS) of the cerebellum and measurements of serum antibodies against TG6. It was discovered that 73% of the patients with an abnormal MRS of the cerebellum, ataxia, or both had anti-TG6 antibodies, indicating that the presence of anti-TG antibodies may be used for screening and diagnosis of gluten ataxia [21].

In terms of diagnosis, it is quite difficult for physicians to detect gluten ataxia, which is usually diagnosed by improvement of symptoms with a gluten-free diet (GFD). An upcoming serological marker could be anti-TG6 antibodies (Table 1) [21]. Radiological investigations include MRS of the cerebellum by measuring N-acetyl aspartate/creatine (NAA/C) area ratios [17]. On MR imaging, 60% of patients with GA reveal cerebellar atrophy [17].

The treatment consists primarily of a GFD. A study conducted in 2017 by Hadjivassiliou et al., included 117 patients with GA who had been diagnosed with the presence of anti-gliadin antibodies, out of which 63 of them were on a strict GFD. It was found that 62 of them expressed increased NAA/C area ratios measured by MRS. The results demonstrate the effectiveness of a GFD for the treatment of GA even in the absence of detectable enteropathy (Table 1) [22]. A new upcoming treatment modality for patients with GFD-resistant GA that is currently being studied is intravenous immunoglobulins [23].

Gluten neuropathy

It is defined as sporadic neuropathy, without apparent etiological causes, with serological evidence of gluten sensitivity, including tissue transglutaminase antibodies, anti-gliadin antibodies, or endomysium antibodies [24]. It occurs in about 23% of the patients with celiac disease (Table 2) [25]. A study conducted by Lousterinen et al., in April 2003 included 26 patients of CD and 23 patients with reflux disease and performed electroneuromyographic tests for tactile, vibrational, and temperature thresholds. It was found that six patients with CD showed signs of chronic axonal neuropathy compared to only one patient with reflux disease. Patients of CD also exhibited significantly higher tactile, heat and pain thresholds in comparison with the patients with reflux disease. This indicates that neurological manifestations occur in CD even in the absence of malabsorption (Table 2) [26]. When it comes to the type of neuropathy, the most common is symmetrical sensorimotor axonal peripheral neuropathy [24]. However, sensory ganglionopathy, small fiber neuropathy, and autonomic neuropathy are also evident [24]. The presence of CD increases the risk for developing neuropathy to 2.5 times compared to the general population [26]. A study conducted in 2008 by Thawani et al., in Sweden included around 160,000 patients, out of which 28,323 were diagnosed with CD by intestinal biopsy and the rest were age and sex-matched controls, and they were assessed for neuropathies. It was found that patients with CD had a 2.5 times higher risk of developing neuropathies (95% CI, 2.1-3.0; P < 0.001). Additionally, there was an increased risk for chronic inflammatory demyelinating neuropathy (2.8; 1.6-5.1; P = 0.001), autonomic neuropathy (4.2; 1.4-12.3; P = 0.009), and mononeuritis multiplex (7.6; 1.8-32.4; P = 0.006) [27]. This extensive study points to the need for screening of patients with CD for neuropathies. However, only one-third of patients with gluten neuropathy have evidence of CD on biopsy [24]. After being diagnosed with CD, the mean duration of onset of neuropathy is nine years, with an average age of 55 years [24]. It is also worth mentioning that CD has no significant correlation with other neurological diseases such as multiple sclerosis, Parkinson's disease, Alzheimer's disease, hereditary ataxia, Huntington's disease, myasthenia gravis, or spinal muscular atrophy [27]. 

**Table 2:** Summary of included studies revealing the clinical characteristics and treatment for gluten neuropathy CD: celiac disease; SNAP: sensory nerve action potential; GFD: gluten-free diet

References	Population	Method	Findings	Conclusion
Lousterinen et al. [25]	Department of Neurology in Tampere University Hospital, Finland	The study included 26 patients with CD and 23 patients with reflux disease.	They performed electroneuromyographic tests for tactile, vibrational, and temperature thresholds. It was found that six patients with CD showed signs of chronic axonal neuropathy compared to only one patient with reflux disease.	CD is associated with neurological manifestations, and they occur in CD even in the absence of malabsorption.
Thawani et al. [26]	Sweden	About 160,000 patients, out of which 28,323 were diagnosed with CD by intestinal biopsy.	They were assessed for neuropathies, and it was found that patients with CD had 2.5 times higher risk.	Patients with CD should be screened for neuropathies.
Hadjivassiliou et al. [30]	Department Neurophysiology, The Royal Hallamshire Hospital, Sheffield, United Kingdom	35 patients with gluten neuropathy diagnosed by serology.	They conducted regular neurological examinations along with the sural SNAP. It was found that adherence to a GFD caused a decrease in the levels of serological markers of gluten sensitivity and an improvement in the neurological symptoms.	GFD can be used as a treatment modality for gluten neuropathy even in the absence of enteropathy.

The mechanism of nerve injury is due to inflammation caused by anti-gliadin antibodies leading to axonal degeneration secondary to an inflammatory vasculopathy similar to gluten ataxia [24,28,29]. HLA types corresponding to CD are present in 80% of the patients with sporadic neuropathy [29]. There is no role of vitamin deficiency due to malabsorption as most of the patients do not have enteropathy. Diagnostic modalities of gluten neuropathy include the presence of anti-gliadin antibodies without any other apparent etiological cause for the neuropathy. Treatment consists primarily of a GFD. A study conducted in 2006 by Hadjivassiliou, included 35 patients with gluten neuropathy, out of which 25 were put on a strict GFD and 10 were not. After following the patients for 10 years (1996-2005) and conducting regular neurological examinations along with sural sensory nerve action potential (SNAP), it was found that adherence to a GFD caused a decrease in the levels of serological markers of gluten sensitivity and an improvement in the neurological symptoms. This indicates that a GFD can be used as a treatment modality for gluten neuropathy even in the absence of enteropathy (Table 2) [30].

Gluten encephalopathy

About 4.4% of the patients with CD also complain of headaches similar to migraines [31]. A study conducted in 2015 Russia by Kopishinskaya and Gustov, included 200 patients with CD and 100 patients without CD. The subjects were asked to keep a headache diary during the course of six months [32]. It was discovered that patients with CD experienced headaches four times more often than the control group (48.5%; p<0.001), and the frequency of the migraine attacks was 2.5 times higher than the control group (р=0.004). In addition, it was found that migraine attacks were more common in patients aged 50 years and above [32]. Another study conducted in Sweden 2016 by Lebwohl et al., included 28,638 patients with CD and 143,126 controls and found that a headache-related visit to the hospital occurred in 4.7% of the patients with CD and 2.9% in the controls. Headaches were evident in patients with enteropathy as well as without enteropathy. This indicates that gluten sensitivity is strongly associated with headaches [33]. It is also worth mentioning that gluten encephalopathy does not present on its own but is usually accompanied by other neurological features such as gluten ataxia and gluten neuropathy [34].

In terms of pathogenesis, there is evidence of white matter abnormalities on MRI [34]. The mechanism seems unclear, but isolated vasculitis has been found on brain biopsy [35]. Other proposed mechanisms include vitamin and mineral deficiencies due to malabsorption [36]. In addition, changes in cerebral blood flow have been observed in patients with CD, which may lead to gluten encephalopathy [37]. A study conducted in 2004 by Addolorato et al., included 54 subjects, out of which there were 30 patients with CD, out of which 15 were on a GFD, and 15 were not. The rest of them were controls. The patients were followed for about one year undergoing regular single-photon emission computed tomography examinations, and it was found that only one patient out of the 15 patients with CD on a GFD developed hypoperfusion in at least one brain region in contrast to the 15 untreated CD patients, out of which 11 developed at least one hypoperfused region in the brain [37]. This study not only highlights the significance of a GFD as a mode of treatment for CD but highlights an altered cerebral blood flow as one of the possible pathological steps for gluten encephalopathy.

Epilepsy

It has been estimated that the prevalence of epilepsy in patients with CD is 5.5% [38]. The most common type is temporal lobe epilepsy (TLE) [38]. A study conducted in 2009 by Peltola et al., included 48 therapy-resistant patients with epilepsy. They were divided into three main groups; 16 patients with TLE with hippocampal sclerosis (HS), 16 with TLE without HS, and 16 with extratemporal epilepsy. All the patients underwent serological testing for gluten sensitivity as well as a duodenal biopsy. It was discovered that seven out of the 16 patients (44%) with TLE with HS were gluten sensitive, in contrast to zero patients being gluten sensitive in the other two groups, and out of the seven, three displayed histological features of CD on duodenal biopsy. This indicates that there may be a strong correlation between TLE with HS and CD [38]. Other areas of the brain that seem to be involved are the occipito-parietal regions [39]. Treatment usually involves anticonvulsants and a GFD [39]. 

## Conclusions

As evident from the studies discussed in this article, there has been a shift in the clinical presentation of CD in the last few decades from being a condition causing malabsorption to an autoimmune condition with multisystem involvement and various clinical presentations in response to gluten ingestion. In summary, the clinical significance of this article was to cover the various neurological manifestations of CD such as gluten ataxia, gluten neuropathy, gluten encephalopathy, and epilepsy. These conditions are strongly associated with a lower quality of life and a higher rate of hospitalization. It is quite challenging for a physician to diagnose CD when a patient presents with neurological symptoms. We believe this article can help inform practicing physicians and spread awareness with regards to the neurological features of CD, which should be kept in mind to raise an adequate amount of clinical suspicion to perform screening for gluten sensitivity so that the time to diagnosis is decreased and patient suffering is kept to a minimum. The magnitude of these problems may be decreased if sufficient screening for neurological damage in patients with CD is carried out. This would require a heightened awareness of gluten sensitivity when a patient presents with idiopathic neurological features. We spoke specifically of the epidemiology of the various neurological manifestations, their possible underlying pathological mechanisms, and the significance of a GFD in their treatment. Finally, we believe that the neurological manifestations of CD require more thorough research studies so that alternative treatment modalities are offered to provide a more holistic treatment approach to a patient with CD.
